# Comparison of Protein Variation in *Protobothrops mucrosquamatus* Venom between Northern and Southeast Taiwan and Association with Human Envenoming Effects

**DOI:** 10.3390/toxins14090643

**Published:** 2022-09-18

**Authors:** Liao-Chun Chiang, Kun-Yi Chien, Hung-Yuan Su, Yen-Chia Chen, Yan-Chiao Mao, Wen-Guey Wu

**Affiliations:** 1 College of Life Sciences, National Tsing Hua University, Hsinchu City 300, Taiwan; 2 Graduate Institute of Biomedical Sciences, College of Medicine, Chang Gung University, Taoyuan County 333, Taiwan; 3 Clinical Proteomics Core Laboratory, Chang Gung Memorial Hospital, Taoyuan County 333, Taiwan; 4 Department of Biochemistry and Molecular Biology, College of Medicine, Chang Gung University, Taoyuan County 333, Taiwan; 5 Department of Emergency Medicine, E-Da Hospital, Kaohsiung County 824, Taiwan; 6 The School of Chinese Medicine for Post Baccalaureate, I-Shou University, Kaohsiung County 840, Taiwan; 7 Department of Safety, Health and Environmental Engineering, National Kaohsiung University of Science and Technology, Kaohsiung County 811, Taiwan; 8 Department of Emergency Medicine, Taipei Veterans General Hospital, Taipei City 112, Taiwan; 9 School of Medicine, National Yang Ming Chiao Tung University, Taipei City 112, Taiwan; 10 Department of Emergency Medicine, National Defense Medical Center, Taipei City 114, Taiwan; 11 Division of Clinical Toxicology, Department of Emergency Medicine, Taichung Veterans General Hospital, Taichung City 407, Taiwan; 12 College of Medicine, National Chung Hsing University, Taichung City 402, Taiwan

**Keywords:** *Protobothrops mucrosquamatus*, inter-population venom variation, snake venom metalloproteinases, phospholipase A_2_, venom-induced blistering

## Abstract

Reports of bite from *Protobothrops mucrosquamatus* (*Pmu*) are frequent in Taiwan, and its wide-spread distribution and diverse habitats drove us to investigate its envenoming effects and relevant venom variations. We used reversed-phase high-performance liquid chromatography and mass spectrometry to analyze 163 *Pmu* venom samples collected from northern and southeastern Taiwan. Twenty-two major protein fractions were separated and analyzed, and their contents were determined semi-quantitatively. The results showed that despite the trivial differences in the protein family, there is an existing variation in acidic phospholipases A_2_s, serine proteinases, metalloproteinases, C-type lectin-like proteins, and other less abundant components in the *Pmu* venoms. Moreover, clinical manifestations of 209 *Pmu* envenomed patients hospitalized in northern or southeastern Taiwan revealed significant differences in local symptoms, such as ecchymosis and blistering. The mechanism of these local effects and possibly relevant venom components were examined. Further analysis showed that certain venom components with inter-population variation might work alone or synergistically with others to aggravate the local effects. Therefore, our findings of the venom variation may help one to improve antivenom production and better understand and manage *Pmu* bites.

## 1. Introduction

*Protobothrops mucrosquamatus* (*Pmu*) is a pit viper widely distributed in southern and southeastern Asia, including north-eastern India, Myanmar, northern Thailand, Laos, Vietnam, southern China, Taiwan, and Japan (Ryukyu islands) [1]. The World Health Organization (WHO) has categorized *Pmu* as the most medically important venomous snake in Taiwan and China [2]. In addition, it is one of the most common snakebite incidents that have led to morbidity or even mortality in Taiwan [3]. As for its envenoming effects on humans, *Pmu* bites result in local tissue swelling; pain; ecchymosis; blistering (bullae formation); wound necrosis; compartment syndrome; and systemic effects, such as thrombocytopenia, coagulopathy, rhabdomyolysis, and renal impairment [4]. However, differences in human envenoming effects between the two regions were not compared in those studies.

The composition of *Pmu* venom is predominately snake venom metalloproteinases (SVMPs) and phospholipases A_2_ (PLA_2_s), followed by snake venom C-type lectin-like proteins (Snaclecs), snake venom serine proteinases (SVSPs), cysteine-rich secretory proteins (CRISPs), and L-amino acid oxidases (LAOs) [5,6]. Since the clinical manifestations of the same species snake bites are closely related to the venom composition, intra-specific variations in the profile and amount of each venom component may have resulted in diversified envenoming effects [7,8,9]. Intra-specific venom variations can be further categorized as intra-population (differences within populations; individual variation) and inter-population (differences between populations) variabilities; such variation is common among pit vipers [10,11].

Recently, two retrospective studies related to clinical manifestations of *Pmu* bites were conducted by two medical centers in Northern and Southeastern Taiwan, respectively [12,13]. Northern and Southeastern Taiwan are separated by the Central Mountain Range (highest peak, 3952 m), and non-alpine snakes, such as *Pmu*, inhabiting these two biogeographical regions can be considered as two distinct populations. In previous studies, a Taiwanese pit viper, *Trimeresurus stejnegeri stejnegeri*, was found to show inter-population venom variations, such as PLA_2_ variation, in the two regions [14,15]. These findings drove us to further investigate inter-population venom variations in *Pmu* venom.

However, the venom variation of *Pmu* remains little understood [14], and its association with the effects of human envenoming have not been studied. Therefore, in this study, we first compared *Pmu* venom components between northern and southeastern Taiwan. Secondly, we examined the clinical manifestations of *Pmu* envenoming between these two regions. We believe that this study can further assist venom and antivenom research and improve the management of *Pmu* bites in Taiwan.

## 2. Results and Discussion

### 2.1. Venom Proteome of Individual Pmu

**Figure 1:**
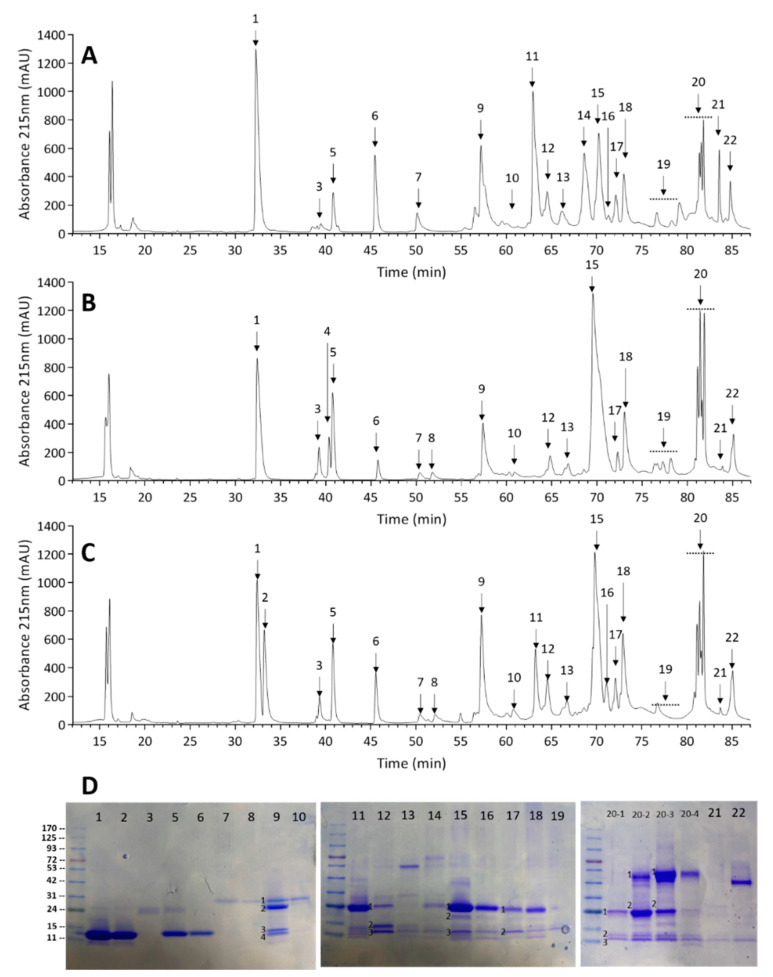
HPLC and SDS-PAGE analyses of *Pmu* venom. In total, 100 µg of the *Pmu* crude venom protein from (**A**) *Pmu001*, (**B**) *Pmu002*, and (**C**) *Pmu003* sample was separated by RP-HPLC; (**D**) each fraction separated in Figure 1C was subjected to the SDS-PAGE analysis under reducing conditions. Molecular mass of the markers (in kDa) are indicated at the gel left.

The individual variation in venom components of 163 adult *Pmu* venoms is shown in Figure 1. Twenty-two protein peaks (labeled Fr 1–22) were identified for most individual *Pmu* venoms in the reverse phase high-performance liquid chromatography (RP-HPLC) profiles (Figure 1A–C). Shotgun proteomics with liquid chromatography tandem mass spectrometry (LC-MS/MS) analysis showed 31 venom components, categorized into nine major protein families: SVMP, PLA_2_, Snaclec, SVSP, CRISP, 5’-nucleotidase (5’-NT), phospholipase B (PLB), glutaminyl-peptide cyclotransferase (QPCT), and LAO. The major venom toxins in each fraction are listed in Table 1. Additionally, detailed proteomics data, including peptide sequences in MS, were provided in the supplementary Table S1. The average amount of the nine protein families of *Pmu* venoms is shown in Table 2. We found that *Pmu* venom contained abundant SVMPs (45.1%) and PLA_2_s (25.0%), followed by Snaclecs (12.8%) and SVSPs (8.23%); however, it contained low contents of 5’-NT (3.85%), LAO (2.37%), CRISP (1.42%), PLB (0.77%), and QPCT (0.46%). These results support the finding of Aird, S. D et al. on Ryukyu *Pmu* venom proteomics, genomcis, and transcriptomes [6,16] and agree with Villalta, M. et al.’s study using pooled *Pmu* venom from Taiwan [5].

### 2.2. Comparison of Inter-Population Variations in Pmu Venom

The inter-population variation in venom protein families of 163 adult *Pmu* venoms (119 patients in northern and 44 in southeastern Taiwan) was compared in Table 2 and Figure S1. No significant difference was found for dominant protein families, such as SVMP (45.95% vs. 42.76%), PLA_2_s (25.56% vs. 23.48%), Snaclec (13.15% vs. 11.86%), SVSP (8.26% vs. 8.15%), and other minor protein families. Even though the difference in the average abundance of venom protein families between the two regions was trivial compared to other pit viper species (Table 2), the details of different protein fractions are worth investigating in order to further understand how they may be related to specific clinical manifestations.

The details of different protein fractions in the venom of individual *Pmu* between populations was compared. The results showed that protein fractions varied between *Pmu* populations from these two regions (Table 3).

**Figure 2:**
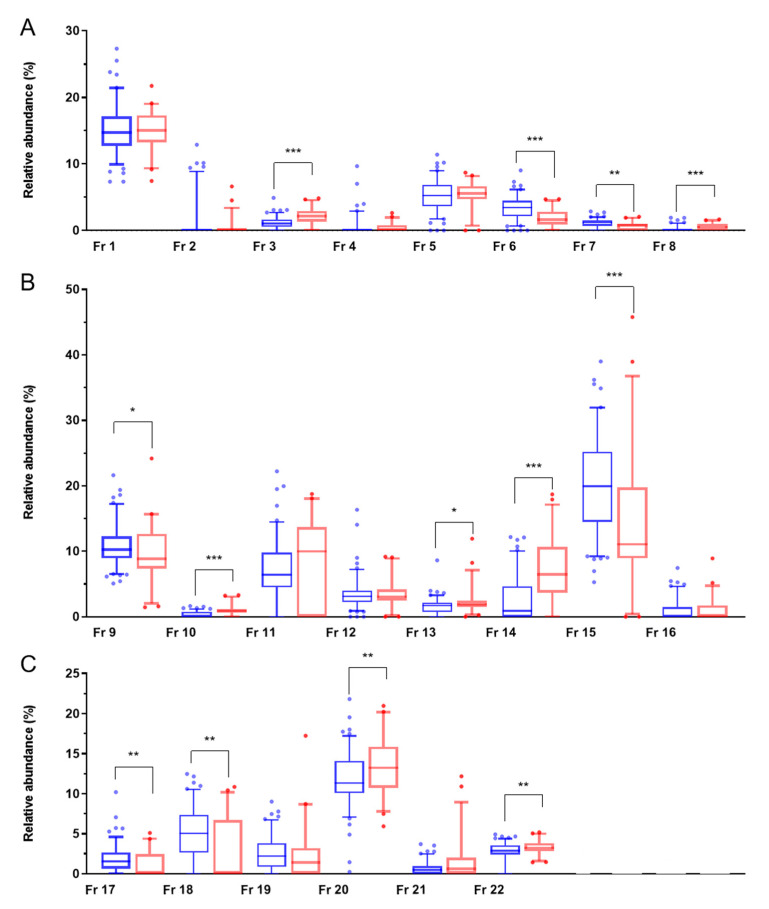
The relative abundances of HPLC-fractions of the 163 *Pmu* venoms in the two geographic regions are shown in (**A**) Fr 1 to 8, (**B**) Fr 9 to 16, and (**C**) Fr 17 to 22; the northern population is marked in blue and the southeastern in red. Box plots showing median (line), 25th–75th percentile (box), and 5th-95th percentile (whiskers) of the individual abundance of the 22 fractions, respectively. Significance of the Mann–Whitney test are denoted (* = *p* < 0.05; ** = *p* < 0.005; *** = *p* < 0.001).

We identified PLA_2_ or its homolog in five major fractions (1, 2, 4, 5, and 6) eluted from HPLC (Table 1). In contrast to the basic PLA_2_ homolog (Fr 1, 2, and 5), which did not show variation, acidic PLA_2_s (Fr 4 and 6) presented a significant variation (Figure 2A, Table 3). The major acidic E6-PLA_2_ (named *Pmu*-PLA-I, with anti-platelet activity) [17] was significantly higher in the venom of the northern *Pmu* population (Fr 6, Figure 2A). Furthermore, another minor acidic R6-PLA_2_ (TmPL-III) was only present in low amounts in some *Pmu* venom from both regions (Fr 4, Figure 2A). Notably, the proportion of *Pmu* venoms containing TmPL-III in the southeastern population was significantly higher than those in the northern region (47.7% vs. 19.3%, *p* < 0.001) (Table 3). However, in a study by Tsai et al., TmPL-III was present in the northern population but absent in the southwestern population in about 20 *Pmu* venom samples [18,19]. Therefore, we suggest that the presence of TmPL-III in *Pmu* venom varies regionally, with more frequent occurrence in the southeastern *Pmu* population. Although there had been claims about the presence of R49-PLA_2_, which was found in Chinese *Pmu*, in Taiwanese *Pmu* from other research [20], we did not find R49-PLA_2_ in our 163 venom samples.

SVMPs derived from the PII-protease domains were the most abundant protein family in all *Pmu* venom samples (Table 2). Proteins of this family were identified from the fraction of 15, 16, 18, 19, 21, 22, and a part of 20 (Table 1). PI-SVMP (Fr 11) and PIII-SVMP (Fr 22) of the *Pmu* samples also showed inter-population variability. Most *Pmu* venoms from the two regions contain PIII-SVMP (Fr 22) (northern area: 93.3%, southeastern area: 100%, Table 3), but the relative abundance of PIII-SVMP was significantly higher in the southeastern area (Figure 2C). Furthermore, a higher proportion of *Pmu* contains PI-SVMP (Fr 11) in the northern region (87.4% vs. 72.7%, *p* = 0.025, Table 3). Additionally, the analytic result of PI-SVMP (Fr 11) is found to be similar to trimerelysin-2 (trimerelysin-2 like) (accession No. P20165) isolated from the venom of *Protobothrops flavoviridis*, which may have no or low hemorrhagic activity [21].

As for SVSP variants, mucrofibrase-3 (Fr 9) was consistently present in venoms from both *Pmu* populations (Table 3), and its relative abundance was similar across the populations (Figure 2B). On the other hand, inter-population variability was found in other SVSPs (Fr 7, 8, and 10). For example, a plasminogen activator similar to TSV-PA (accession No. Q91516, of *T. s. stejnegeri* venom) was identified in Fr 7. Its relative abundance (Figure 2A) and proportion of presence (82.4% vs. 65.9%, *p* = 0.025, Table 3) was significantly higher in the northern population. As for Snaclecs, trimecetin identified from Fr 15 and 17 was more prevalent, with higher relative abundance in the northern population (Table 3, Figure 2C). Both LAO enzymes (accession No. T2HRS5) (Fr 13 and 20) and 5’-NT (Fr 14) were more frequent and abundant in the venom of the southeastern population (Table 3, Figure 2B).

In short, we disclosed that the major variations were acidic PLA_2_ variants (i.e., E6- and R6-PLA_2_), Snaclec, and metalloproteases of PII-class by analyzing many individual *Pmu* venoms (Table 3, Figure 2). To produce effective antivenom, guidelines recommended that an adequate number of individual snakes from various regions covering the entire geographic distribution of specific venomous snake species should be collected together [2]. Our findings may help evaluate the pooled snake venoms based on characters of inter-population variation and potentially assist in improving antivenom titer against snake venom from these regions.

### 2.3. Regional Variation in the Clinical Manifestations of Pmu Envenoming

A total of 209 *Pmu* envenoming cases, in 149 and 60 patients from northern and southeastern Taiwan, respectively, were compared to investigate the regional variation in clinical manifestations (Table 4). A higher proportion of the *Pmu* envenoming cases in northern Taiwan developed ecchymosis (75.2% vs. 21.7%, *p* < 0.001) and blistering (17.5% vs. 5%, *p* = 0.019). No significant difference was found for local complications, such as tissue necrosis (11.4% vs. 5%, *p* = 0.154) and compartment syndrome (6% vs. 8.3%, *p* = 0.549), or in the systemic complications. There were slightly higher proportions of cellulitis (25.5% vs. 13.3%, *p* = 0.055) and renal impairment (12.3% vs. 5.4%, *p* = 0.088) in patients from the northern region than those from southeastern area.

Currently, there are only a few studies that examined the difference in snake envenoming effects regarding the geographic locations available, with most of them focusing on the comparison of the severity score and antivenom dosage following bites between regions [7,8,9]. Our study is the first to investigate the clinical manifestation differences of single species snakebite in northern and southeastern Taiwan. The variation in clinical effects is likely to be affected by the variation in venom components, as suggested in two case studies of *Crotalus pyrrhus* bites in the western and eastern regions of Arizona state in America, respectively [9]. Although specific antivenom dosages used for envenomed patients in the northern and southeastern Taiwan differed (with the mean of 4.4 vials for northern region and 6.4 vials for southeastern area), a solid conclusion for differences in antivenom usage could not be simply drawn based on this data. Thus, further antivenomic evaluation of the neutralization effect on major toxic venom components will be essential for the determination of antivenom administration in managing *Pmu* bites.

### 2.4. Possible Venom Components Contributing to Regional Differences in Clinical Manifestations

Since there were significant differences in ecchymosis and blistering with the high similarity shown in venom components, the results are worthy of further discussion. Ecchymosis is a local hemorrhage near the skin’s surface caused by mechanical injury of the capillary basement membrane and is found to be more common in *Pmu* envenoming of northern victims (Table 4). SVMPs are considered as the main components in viperid venom that contribute to hemorrhage [22]. SVMPs degrade extracellular matrix proteins in the capillary basement membrane, causing endothelial cell apoptosis [23,24,25] and inducing hemorrhage [26]. P-II and PIII-metalloproteases bind more specifically to the capillary basement membrane than PI-SVMPs, causing higher hemorrhagic activity [26,27,28]. Furthermore, the non-catalytic PLA_2_ (myotoxic and cytotoxic [29,30]) in viperid venom could induce endothelial cell detachment synergistically with PIII-SVMP [31] and possibly enhance myotoxicity caused by non-catalytic SVSP [32]. Based on the biochemical effects of enzymes and our findings of the variation in venom components and clinical manifestations, we suggest that a higher incidence of venom-induced ecchymosis in patients from the northern region is likely to be caused by PI-SVMP (Fr 11) and PII-SVMP (Fr 15, 18, and 19).

Venom-induced blistering, characterized by the separation of the epidermis and dermis [33], is a clinical feature more frequently observed in patients from northern Taiwan (Table 4). SVMPs, particularly PI-SVMPs, induce the proteolytic cleavage of skin basement membrane components at the dermal–epidermal junction [34,35,36,37,38]. Additionally, the proteolytic action of endogenous matrix metalloproteinases in local tissues activated by inflammatory reactions may be involved in blister formation [37,38]. Moreover, the activation of the complement system induced by PI-SVMPs promotes local tissue inflammation [39]. K49-PLA_2_ induces cell membrane damage through a non-catalytic mechanism and release inflammatory mediators in tissues [30,40,41,42]. Catalytic D49-PLA_2_s enhance the release of inflammatory mediators synergistically with K49-PLA_2_ [29,30]. The high abundance of PI-SVMP (trimerelysin-2 like), PII-SVMP (PMMP-3), and acidic E6-PLA_2_ in the northern *Pmu* venom may jointly attribute to more severe local symptoms. Therefore, this information could be important for antivenom manufacturers and clinicians in assessing antivenom efficacy and effectiveness in managing *Pmu* bites. Although the differences of minor components (i.e., Snaclec, CRISP, LAO, PLB, QPCT, and 5’-NT) in venoms were found between regions, their clinical significance in *Pmu* envenoming remains undetermined [43,44,45,46]. Furthermore, a proper-designed animal model examining the ecchymosis and blistering effect by the subcutaneous injection of venom component at different doses may be helpful in the determination of the causal relationship between the venom component and the associated clinical effect.

### 2.5. Limitations

Although we found inter-population variations in venom compositions and suggested the associations of venom variations with clinical manifestations, this study has some limitations. First, the body size, age, and sex of individual snakes; the amount of venom injection; and the health status of patients, which are important factors that may have affected the severity and treatment of envenomation, are difficult to determine in such retrospective studies. Second, it is challenging to separate and analyze snake venom components by RP-HPLC with gel electrophoresis because of their similar molecular weights or hydrophobicity. Furthermore, the method used for protein resolution or separation (mainly re-dissolved or reconstituted venom solution) might cause certain non-protein loss and precipitation of high-molecular-weight constituents, making these components undetectable. Third, the bottom-up venomomics applied in this study may provide incomplete compositional information of venoms, especially for low-abundance components. The top-down mass venomics may eliminate the shortcomings of bottom-up workflows [47,48]. Finally, the data of this study were extracted from medical charts that cover a long period. This introduces the possibility of variations in how the information has been introduced into the charts; that is, different physicians may introduce a different degree of detail in the charts. Additionally, different physicians were likely involved in the therapeutic decisions of these patients. Since this is a retrospective study and has its inherent limitations, results should be interpreted cautiously. Nevertheless, our pioneering study on the investigation of the variation in *Pmu* venoms and clinical envenoming effects in different regions in Taiwan has strong potential in improving therapeutic implications in snakebite management.

## 3. Conclusions

We found that there is inter-population variability in the presence of *Pmu* venom protein fractions 4, 7, 8, 10, 11, 13, 14, 17, 18, and 19 (i.e., PLA_2_ TmPL-III, SVSP, KN-SVSP, SVSP, PI-SVMP, LAO, 5’-NT, Snaclec, PII-SVMP, and PII-SVMP, respectively). A higher proportion in fractions 7, 11, 17, 18, and 19 (i.e., SVSP, PI-SVMP, Snaclec, PII-SVMP, and PII-SVMP, respectively) was noticed in the northern snakes’ population. Additionally, the amount of relevant protein families is higher in fractions 3, 6, 7, 8, 10, 13, 14, 15, 17, 18, 20, and 22 in the northern snakes’ population even though the presence of certain fractions (i.e., 6, 15, and 20) does not differ between regions.

Furthermore, we found a higher risk of developing ecchymosis and blistering in the northern patients’ population, which is suggested to be associated with the higher abundance of PI-SVMP (trimerelysin-2 like), PII-SVMP (PMMP-3), Snaclec (Trimecetin), SVSP (TSV-PA like), and acidic E6-PLA_2_ in the *Pmu* venoms obtained from the same areas. Our study provides objective information on *Pmu* venoms, and these findings can have therapeutic implications for future antivenom manufacturing processes, efficacy evaluation against these major fractions, and snakebite management in Taiwan.

## 4. Materials and Methods

### 4.1. Snake Venoms

Venom samples of 163 adult *Pmu* were obtained from two regions: Taipei Basin and the surrounding mountain areas in northern Taiwan (*n* = 119) and East Rift Valley in southeastern Taiwan (*n* = 44) (Figure 1). Each individual *Pmu* was manually restrained while venom was milked from both half-erected fangs into a 1.5 mL Eppendorf tube. The fresh yellowish crude venom was lyophilized and stored at –20 °C.

### 4.2. Reversed-Phase High-Performance Liquid Chromatography (RP-HPLC)

Individual *Pmu* crude venom was reconstituted in ultrapure water containing 0.1% trifluoroacetic acid (TFA, Sigma-Aldrich, MO, USA) and 5% acetonitrile (Mallinckrodt Baker, NJ, USA) and centrifuged at 10,000× *g* for 10 min. The protein concentration of the supernatant was determined using the Pierce bicinchoninic acid (BCA) protein assay kit (Thermo Fisher Scientific, IL, USA). Venom proteins were separated by RP-HPLC following the method of Villalta, M. et al. study [5]. Briefly, 100 μg of venom protein in 10 μL ultrapure water containing 0.1% TFA (solution A) was injected into an ultra-pressure liquid chromatography system (LC-20ADXR, Shimadzu, Kyoto, Japan) equipped with a diode-array detector (SPD-M20A, Shimadzu, Kyoto, Japan). Chromatography was performed using a Phenomenex Jupiter C18 (250 × 4.6 mm, 5 µm particle size, 300 Å pore size) column at 1 mL/min with a linear gradient of 5% solution B (0.1% TFA in acetonitrile) for 5 min, 5–25% solution B for 10 min, 25–45% solution B for 60 min, and 45–70% solution B for 10 min. The effluent was monitored at 215 nm, and eluted peaks were manually collected and lyophilized.

No individual venom samples in this study have all the HPLC-resolvable protein fractions. However, we have identified as many fractions as possible. The isolated fractions were collected and analyzed from representative samples, including *Pmu*001 (collected from East Rift Valley), *Pmu*002 (collected from Taipei Basin), and *Pmu*003 (collected from Taipei Basin). *Pmu*003, containing the most resolvable fractions, was chosen as the primary reference (Figure 1D). Along with unique fractions obtained from two other samples, all resolvable fractions were subjected to subsequent sodium dodecyl sulfate-polyacrylamide gel electrophoresis (SDS-PAGE) and tandem mass spectrometry (MS/MS) to identify the proteins.

### 4.3. SDS-PAGE and LC-MS/MS Analysis

Before gel electrophoresis, venom samples in 20 μL Laemmli sample buffer containing 25 mM dithiothreitol (Biosynth AG, Switzerland) were boiled for 5 min and alkylated with 135 mM iodoacetamide (Amersham Biosciences, UK). Then, venom proteins were separated through SDS-PAGE, and the gel was stained with Coomassie brilliant blue. Further preparation of MS samples was conducted by cutting each stained band into a few slices, which were destained in 10% methanol (Mallinckrodt Baker, NJ, USA), dehydrated in acetonitrile, and dried in a SpeedVac. Proteins in the gel were digested by sequencing-grade modified porcine trypsin (20 μg/mL; Promega, Madison, WI, USA) overnight at 37 °C. Then, the tryptic peptides were extracted with 200 μL of 0.1% formic acid (Sigma-Aldrich, MO, USA) and 50% acetonitrile solution, with 70% acetonitrile in ultrapure water, dried in SpeedVac, and finally stored at −80 °C for subsequent processing.

Peptide mixtures were reconstituted with 0.1% formic acid and analyzed by LC-MS/MS using an LTQ Orbitrap Velos mass spectrometer coupled with a nano-LC (Thermo Fisher, San Jose, CA, USA). Further, the sample was loaded into a trap column (Zorbax 300SB-C18, 0.3 × 5 mm; Agilent Technologies, Wilmington, DE) at a flow rate of 0.2 μL/min in solution A (0.1% formic acid in ultrapure water) and separated on a resolving 10-cm analytical C18 column (inner diameter, 75 μm) using a 15-μm tip (New Objective, Woburn, MA, USA). The peptides were eluted using a linear gradient of 0–10% solution B (0.1% formic acid in acetonitrile) for 3 min, 10–30% solution B for 35 min, 30–35% solution B for 4 min, 35–50% solution B for 1 min, 50–95% solution B for 1 min, and 95% solution B for 8 min, with a flow rate of 0.25 μL/min. The Orbitrap’s resolution was set at 30,000, and the ion signal of (Si(CH_3_)_2_O)_6_H^+^ at 445.120025 (*m*/*z*) was used as a lock mass for internal calibration.

For MS scan, the *m*/*z* value of scan range was 400–2000 Da. For MS/MS scan, more than 1 × 10^4^ ions were accumulated in the ion trap, and the *m*/*z* values selected were dynamically excluded for 180 s. Both MS and MS/MS spectra were acquired using one scan with maximum 1000 and 100 ms fill-times, respectively. MS raw data were analyzed using Proteome Discoverer Software (v.1.4.1.14; Thermo Fisher, San Jose, CA, USA) and searched against a non-redundant Swiss-Prot database of Serpents (downloaded in August 2021, 273,008 entries) using MASCOT. Search parameters for tryptic peptides included one missed cleavage, a mass tolerance of 0.5 Da for fragment ions, fixed cysteine modification with carbamidomethylation, variable methionine oxidation, variable N-terminal acetylation, and variable transformation of N-terminal glutamate or glutamine to pyroglutamic acid. Finally, the criteria used for protein identification were high peptide confidence; the lowest peptide length was five amino acids, and the minimal number of peptides per protein was two.

### 4.4. Relative Abundance of Protein Families

The relative content of each major protein family was estimated as described by Calvete et al. [49]. Then, the relative content of each LC-eluted protein-peak was calculated based on its peak area using LabSolution (v.5.93, Shimadzu, Kyoto, Japan). For venom protein presenting more than one band in SDS-PAGE, the relative abundance of each protein was estimated by densitometry using GelQuantNET (BiochemLabSolutions Corp., USA). Finally, the abundance of each protein family was calculated as the percentage of the whole venom proteome.

### 4.5. Clinical Manifestations of Pmu-Envenomed Patients in Two Taiwan Regions

**Figure 3:**
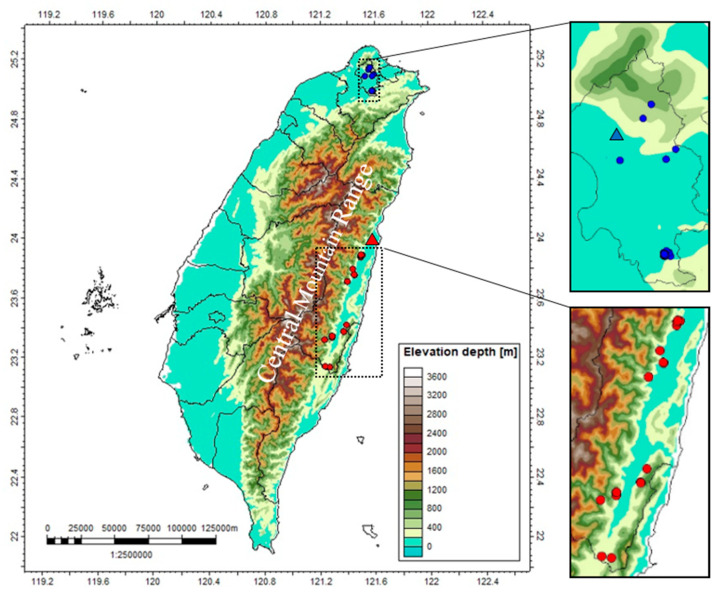
The collection locales of *Pmu* venom of northern (blue dots) and southeastern (red dots) populations, and the locales of Taipei Veterans General Hospital (blue triangle) and Hualien Tzu Chi Hospital (red triangle).

This is a secondary analysis of the retrospective cohort studies [12,13]. All patients enrolled were admitted to two medical centers, Taipei Veterans General Hospital (1991–2006) in northern Taiwan and Hualien Tzu Chi Hospital (2008–2013) in southeastern Taiwan (Figure 3). The culprit snake was identified by examining the snake brought-in by the patient or identifying the snake in a standard picture exhibited in the emergency department [12,13]. The case was excluded in the study if the snake could not be identified [13].

**Figure 4:**
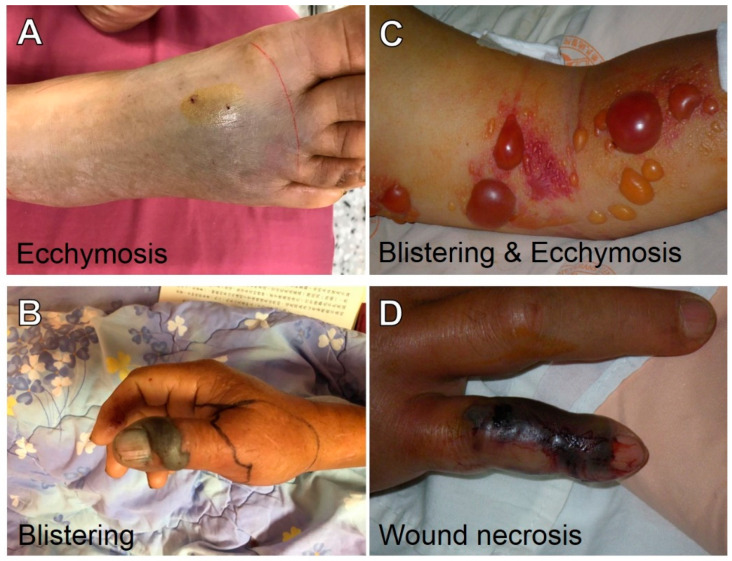
Local effects of *Pmu* envenoming. (**A**) Ecchymosis; (**B**) blistering; (**C**) blistering on the skin; and (**D**) local necrosis on an envenomed finger.

The clinical data collected included patients’ clinical presentation, laboratory analyses, clinical outcomes, and complications. The clinical manifestations of *Pmu* envenomation were classified into three categories: (1) local symptoms, including ecchymosis (Figure 4A,B) and blistering (bulla formation) (Figure 4B,C); (2) local complications, including cellulitis, wound necrosis (Figure 4D), and compartment syndrome; (3) systemic complications, including thrombocytopenia (with platelet count < 150 × 10^9^/L), coagulopathy (with an international normalized ratio >1.25), renal impairment (with serum creatinine level >1.4 mg/dL), and rhabdomyolysis (with blood creatine kinase level >1000 U/L). The study protocols were approved by the Institutional Review Board of Taipei Veterans General Hospital (95-02-25A) and Hualien Tzu Chi Hospital (IRB102-38).

### 4.6. Statistical Analysis

The normality of distribution of continuous variables was tested by one-sample Kolmogorov–Smirnov test. Continuous variables with normal distribution were compared using independent samples Student’s *t*-test and presented as mean ± standard deviation (SD); non-normal variables were compared by the Mann–Whitney U test reported as the median interquartile range (IQR). Categorical variables were compared using the Chi-square test or Fisher’s exact test. We analyzed all the data using the Statistical Package for the Social Sciences v.21.0 (released 2012; IBM Corp., Armonk, NY, USA). A two-tailed *p*-value of <0.05 was considered statistically significant.

## Figures and Tables

**Table 1:** Assignment of the HPLC-fractions of *Pmu* venom based on MS/MS study.

Frcation	Protein Name ^a^	Protein Family	Species	Accession No.
1	basic PLA_2_ homolog, TMV-K49	PLA_2_	*Pmu*	P22640
2	basic PLA_2_ homolog	PLA_2_	*Pmu*	~P22640
3	cysteine-rich venom protein, TM-CRVP	CRISP	*Pmu*	P79845
4	acidic R6-PLA_2_, TmPL-III	PLA_2_	*Pmu*	Q9I968
5	basic N6-PLA_2_, Trimucrotoxin	PLA_2_	*Pmu*	Q90W39
6	acidic E6-PLA_2_, TmPL-I	PLA_2_	*Pmu*	Q91506
7	plasminogen activator, TSV-PA	SVSP	*T. stejnegeri*	Q91516
8	kallikrein-CohID-4	SVSP	*C. o. helleri*	T1DMM6
9	beta-fibrinogenase mucrofibrase-3	SVSP	*Pmu*	Q91509
9	trimecetin-beta subunit	Snaclec	*Pmu*	Q5FZI5
10	beta-fibrinogenase mucrofibrase-2	SVSP	*Pmu*	Q91508
11	trimerelysin-2	PI-SVMP	*P. flavoviridis*	P20165
12	mucrocetin-alpha subunit	Snaclec	*Pmu*	Q6TPH0
12	trimecetin-beta subunit	Snaclec	*Pmu*	Q5FZI5
13	L-amino acid oxidase	LAO	*P. flavoviridis*	T2HRS5
14	ecto-5’-nucleotidase	5’-NT	*P. elegans*	A0A077L6M5
15	Zn-metalloproteinase, PMMP-3	PII-SVMP	*Pmu*	E9NW28
15	trimecetin-beta subunit	Snaclec	*Pmu*	Q5FZI5
16	Zn-metalloproteinase, PMMP-3	PII-SVMP	*Pmu*	E9NW28
17	trimecetin-beta subunit	Snaclec	*Pmu*	Q5FZI5
18	Zn-metalloproteinase, PMMP-3	PII-SVMP	*Pmu*	E9NW28
19	Zn-metalloproteinase, PMMP-3	PII-SVMP	*Pmu*	E9NW28
20	Zn-metalloproteinase, TM-3	PII-SVMP	*Pmu*	O57413
20	trimecetin-alpha subunit	Snaclec	*Pmu*	Q5FZI6
20	trimecetin-beta subunit	Snaclec	*Pmu*	Q5FZI5
20	phospholipase B-like	PLB	*Pmu*	A0A1W7RER1
20	glutaminyl cyclotransferases	QPCT	*Pmu*	M9ND11
20	L-amino acid oxidase	LAO	*P. elegans*	A0A077L6L4
21	Zn-metalloproteinase, PMMP-1	PII-SVMP	*Pmu*	E9NW26
22	Zn-metalloprotease P-IIIa (Fragment)	PIII-SVMP	*P. elegans*	A0A077L7D6
22	flavorase	PIII-SVMP	*P. flavoviridis*	G1UJB2

^a^ abbreviations: PLA_2_, phospholipases A_2_; PI-, PII-, PIII-SVMP, PI-, PII-, or PIII-class of venom metalloproteinase; Snaclec, snake venom C-type lectin like proteins; SVSP, snake venom serine protease; LAO, L-amino acid oxidase; CRISP, cysteine-rich secretory protein; 5’-NT, 5’-nucleotidase; PLB, phospholipase B; and QPCT, glutaminyl-peptide cyclotransferase.

**Table 2:** Relative abundance of the venom proteins of individual *Pmu* in the two geographic regions presents by mean value.

Protein Family ^a^	Northern	Southeastern	Total	*p*-Value
Sample number	*n* = 119	*n* = 44	*n* = 163	
SVMP, total	45.95	42.76	45.1	0.001
-PI or PII class	43.09	39.34	42.11	<0.001
-PIII class	2.86	3.42	3.01	0.011
PLA_2_	25.56	23.48	25.0	0.033
SVSP	8.26	8.15	8.23	0.364
Snaclec	13.15	11.86	12.8	0.01
CRISP	1.11	2.25	1.42	<0.001
LAO	2.15	2.97	2.37	0.01
5’-NT	2.63	7.15	3.85	<0.001
PLB	0.74	0.86	0.77	0.172
QPCT	0.44	0.52	0.46	0.05

^a^ abbreviations are the same as in Table 1.

**Table 3:** Presence of the HPLC-fraction in *Pmu* venom obtained from the two geographic regions.

Fraction	Northern, *n* (%) ^a^				Southeastern, *n* (%)				Protein Family	*p*-Value
1	119	(	100	)	44	(	100	)	PLA_2_	1
2	17	(	14.3	)	2	(	4.55	)	PLA_2_	0.103
3	95	(	79.8	)	39	(	88.6	)	CRISP	0.192
4	23	(	19.3	)	21	(	47.7	)	PLA_2_ TmPL-III	<0.001
5	116	(	97.5	)	42	(	95.5	)	PLA_2_	0.612
6	115	(	96.6	)	40	(	90.9	)	PLA_2_	0.213
7	98	(	82.4	)	29	(	65.9	)	SVSP	0.025
8	27	(	22.7	)	25	(	56.8	)	KN-like SVSP	<0.001
9	119	(	100	)	44	(	100	)	SVSP, Snaclec	1
10	56	(	47.1	)	37	(	84.1	)	SVSP	<0.001
11	104	(	87.4	)	32	(	72.7	)	PI-SVMP	0.025
12	116	(	97.5	)	42	(	95.5	)	Snaclec	0.612
13	94	(	79.0	)	43	(	97.7	)	LAO	0.003
14	74	(	62.2	)	39	(	88.6	)	5’-NT	0.001
15	119	(	100	)	42	(	95.5	)	PII-SVMP, Snaclec	0.072
16	59	(	49.6	)	17	(	38.6	)	PII-SVMP	0.214
17	93	(	78.2	)	18	(	40.9	)	Snaclec	<0.001
18	100	(	84.0	)	21	(	47.7	)	PII-SVMP	<0.001
19	97	(	81.5	)	29	(	65.9	)	PII-SVMP	0.035
20	119	(	100	)	44	(	100	)	SVMP, others ^b^	1
21	78	(	65.6	)	30	(	68.2	)	PII-SVMP	0.752
22	111	(	93.3	)	44	(	100	)	PIII-SVMP	0.109

^a^ Values in parentheses are % of the 119 (Northern) or 44 (Southeastern) *Pmu* samples containing the specific fraction. ^b^ Snaclec, PLB, LAO, and QPCT.

**Table 4:** Comparison of the clinical manifestations of *Pmu* envenomed patients between the two geographic regions.

Region	Northern				Southeastern				*p*-Value
Patient number	*n* = 149	(	%	)	*n* = 60	(	%	)	
Local symptoms									
Ecchymosis	112	(	75.2	)	13	(	21.7	)	<0.001
Blistering	26	(	17.5	)	3	(	5	)	0.019
Local complications									
Cellulitis	38	(	25.5	)	8	(	13.3	)	0.055
Tissue necrosis	17	(	11.4	)	3	(	5	)	0.154
Compartment syndrome	9	(	6	)	5	(	8.3	)	0.549
Systemic complications									
Thrombocytopenia	18	(	12.1	)	8 (*n* = 57)	(	14	)	0.705
Coagulopathy	9	(	6	)	2 (*n* = 58)	(	3.5	)	0.455
Acute renal impairment	8	(	5.4	)	7 (*n* = 57)	(	12.3	)	0.088
Rhabdomyolysis	17	(	11.4	)	3 (*n* = 27)	(	5.1	)	0.964

## Data Availability

Not applicable.

## Supplementary Materials

The following are available online at https://www.mdpi.com/article/10.3390/toxins14090643/s1, Figure S1: Relative abundance of the protein families of individual *Pmu* venom in the two geographic regions.
