# Supplementary material for: Comparison of Protein Variation in Protobothrops mucrosquamatus Venom between Northern and Southeast Taiwan and Association with Human Envenoming Effects
Source: Toxins (Basel). 2022 Sep 18;14(9):643. doi: 10.3390/toxins14090643 (PMC9501293; doi:10.3390/toxins14090643)
Supplement: Supplementary file 1 [file toxins-14-00643-s001.zip › toxins-1836912-supplementary.pdf]

toxins

MDPI

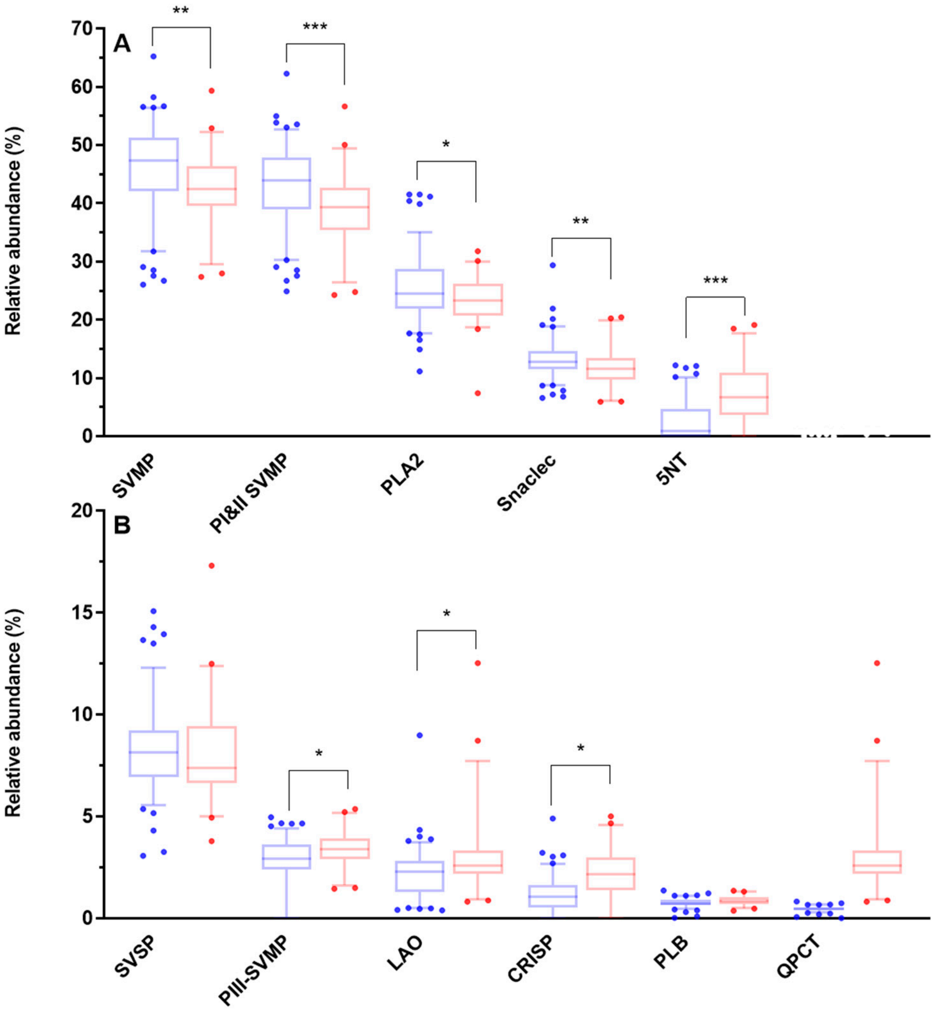

Figure S1. Relative abundance of the protein families of individual *Pmu* venom in the two geographic regions; the northern population is marked in blue and the southeastern in red. Box plots showing median (line), 25th–75th percentile (box) and 5th-95th percentile (whiskers) of the individual abundance of the 22 fractions, respectively. Significance of the Mann-Whitney test are denoted (* = $p < 0.05$; ** = $p < 0.005$; *** = $p < 0.001$).
